# Potential therapeutic uses of intraoral mesenchymal stem cells in other tissues of the body: A review

**DOI:** 10.4317/jced.56809

**Published:** 2021-03-01

**Authors:** Valentina Villarroel, Pascale Fagalde, David Reininger

**Affiliations:** 1 DDS, Private Practice; 2 DDS, PhD, Master in Oral Surgery and Implantology, Assistant Professor, Universidad Mayor, Santiago, Chile

## Abstract

**Background:**

Over the last few years, there has been a great advance in regenerative medicine, with various studies that have observed the ability to repair or regenerate dysfunctional tissues with the patient’s own cells, such as with mesenchymal cells. In this area, mesenchymal stem cells (MSCs) from the oral cavity have attracted attention because of their easy access and multiple cellular differentiations. Multiple studies have shown the various clinical applications at the intraoral level, especially at the level of bone regeneration, but the potential applications of oral MSC at a systemic level have been scarcely described. Objective: The objective of this review was to describe the potential therapeutic uses of intraoral MSCs in other tissues of the organism.

**Material and Methods:**

A review of the literature between 2000 and 2019. Only included those studies done on animals or humans.

**Results:**

Twenty five articles were selected, all performed on animals. The donor site most used were the temporary teeth exfoliated from humans, representing 56% of the total articles, followed by the dental pulp with 28% of the total articles included. Transplantation of intraoral mesenchymal cells in animals with neural tissue illness was the most studied therapy.

**Conclusions:**

Although obtaining MSC of intraoral origin has proven to be a good alternative in regenerative medicine, achieving therapeutic uses in bone tissue, nervous tissue, liver tissue, skin tissue, ocular tissue, reperfusion of tissues and in autoimmune diseases, there is a lack of clinical studies that allow its safe use in humans.

** Key words:**Mesenchymal stem cells, stem cell transplantation, regenerative medicine, dental component.

## Introduction

Regenerative medicine is a multidisciplinary field that through stem cells, scaffolds and growth factors seeks to repair, regenerate or replace tissues, cells or organs that have been damaged. Stem cells are undifferentiated cells capable of self-renewing to replicate themselves, giving rise to specialized cells under appropriate conditions. There are several types of stem cells, among which we find the mesenchymal stem cells (MSC) (1).

The MSC are multipotential adult cells, which fulfill the function of maintaining and repairing the cell population. The characteristics of the MSC is that they have immunomodulatory and immunosuppressive properties altering the innate and adaptive immune response of the individual, in addition, they are able to pass unnoticed in front of the immune system having the potential to be used in therapeutic roles (1,2).

In addition, MSC are able to migrate and graft into damaged tissues where they exert functional and local effects; secrete bioactive molecules such as growth factors, cytokines and chemokines which allow them to have antiapoptotic effects and the stimulation of tissue regeneration (2) and finally, they have plasticity, which is defined as the ability of a cell to differentiate into mature cells different from those of its tissue of origin. This means that they are able to differentiate not only in cells of the mesoderm, but also in cells of the endoderm and ectoderm (1). All these properties of MSC have called attention to use them for therapeutic purposes in tissues or organs damaged in the life of different individuals.

Currently it has been shown that MSC are possible to find in multiple sites of the organism, with MSC coming from the bone marrow the main source of obtaining this type of cells and at the same time they have been the most studied, so they are the apparent gold standard established (2).

However, a medical team is necessary for general or epidural anesthesia to be obtained, where there is also postoperative pain, morbidity risks for the patient and it is considered an invasive procedure when compared to the extraction of stem cells from other parts. of the body. As a result of the disadvantages presented by the bone marrow as a source of MSC, other alternatives of donor sites have been sought, in order to obtain them in a minimally invasive manner (3).

In the oral cavity, unlike other MSC donor sites it is possible to obtain them in a simple and non-invasive way, which are found in the dental pulp (DPSCs), periodontal ligament (PLSCs), apical papilla (SCAP), temporal teeth exfoliated (SHED), dental follicle (DFSCs), periosteum, dental germ (GDSCs), gingival tissue, salivary glands, adipose tissue (ASC) and alveolar bone (3,4).

It has been observed that intraorally these cells have achieved bone regeneration, tooth root regeneration, pulp regeneration, regeneration of the dentin-pulp complex, regeneration of the periodontal ligament and regeneration of periodontal defects (2,4). However, there’s still no certainty of the use that has been granted to this type of cells at a systemic level. Therefore, the objective of this research is to describe the potential therapeutic uses of MSCs of intraoral origin in other tissues of the organism.

## Material and Methods

A review of the literature was carried out between 2000 and 2019 in the databases PubMed, EBSCO and Google Scholar, the details of the search methodology can be found in Table 1. Studies animals or in humans were included, studies that used intraoral MSC in damaged tissues outside the stomatognathic system, randomized and non-randomized clinical trials, cohort studies, case-control studies and case reports that were published between the year 2000 to February 2019 in English or Spanish. In vitro and ex vivo studies, narrative reviews, studies that did not specify the MSC used, studies that included animals or humans in pharmacological therapy that affected tissue regeneration, studies that induced immunosuppression or used immunocompromised animals or humans were excluded.

**Table 1:**
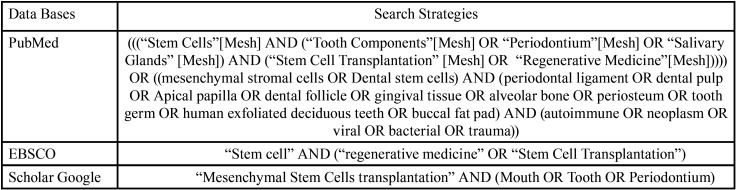
Search strategy.

## Results

**Figure 1:**
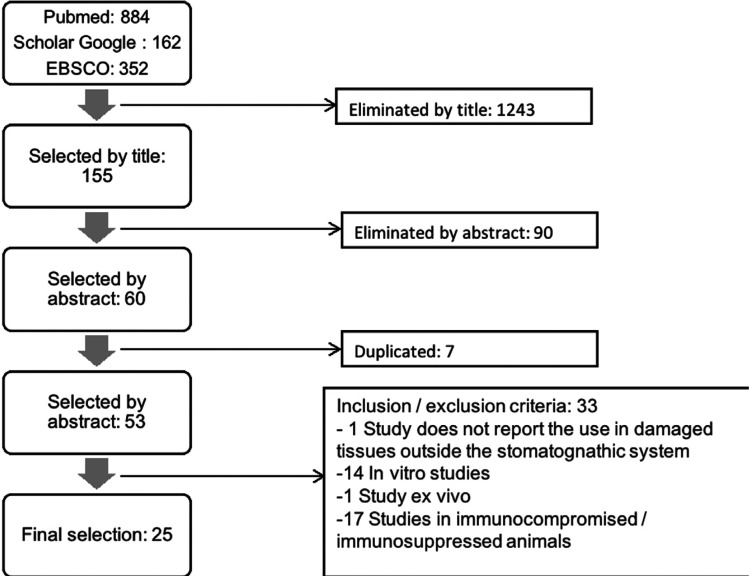
Articles selection.

As a result of the electronic research, a total of 1398 articles were found, of which, by eliminating according to the inclusion and exclusion criteria, a total of 25 articles were selected, as summarized in Figure 1. All articles found in this review belong to studies carried out on animals (Table 2 summarizes the selected articles,  Table 2 cont.).

**Table 2:**
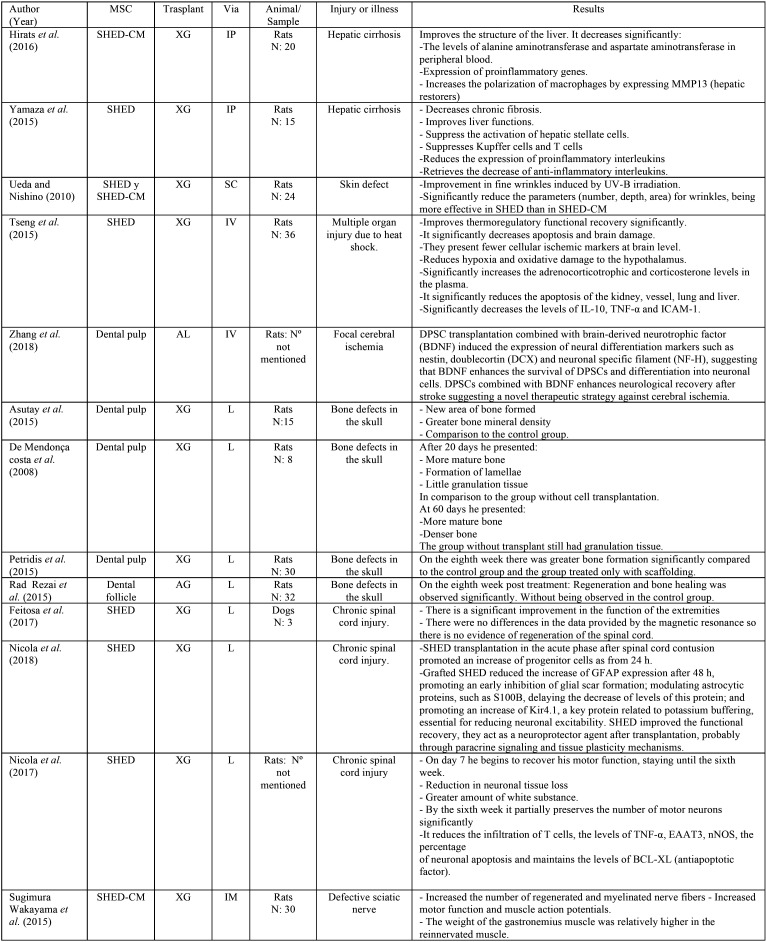
Summary of the included studies.

**Table 2 cont.**
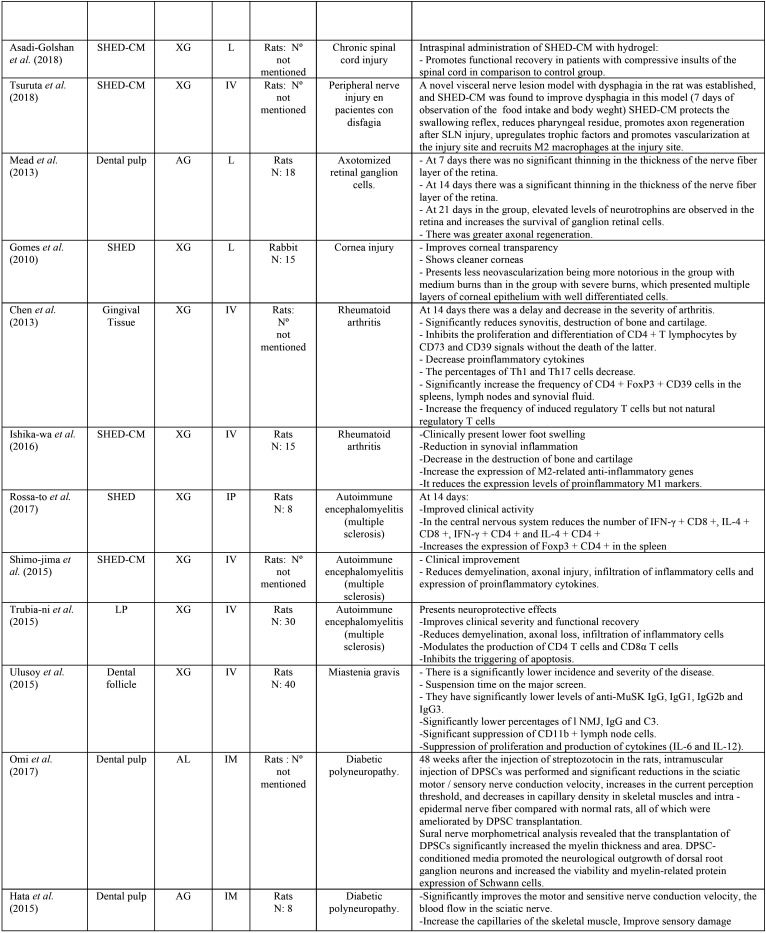
Summary of the included studies.

**Figure 2:**
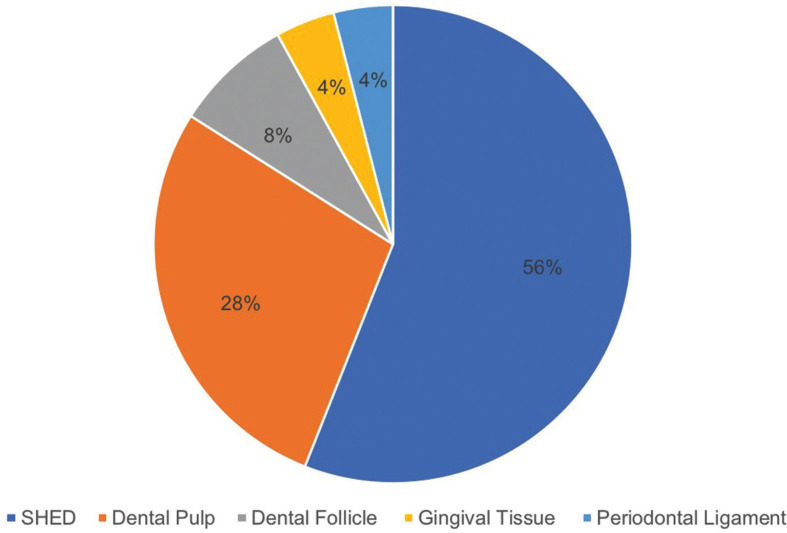
Intraoral donor sites of MSC.

Within the intraoral MSC donor sites used in the selected studies, the most used was SHED in a 56% of the articles, followed by DPSCs in a 28% of the studies (Fig. 2).

**Figure 3:**
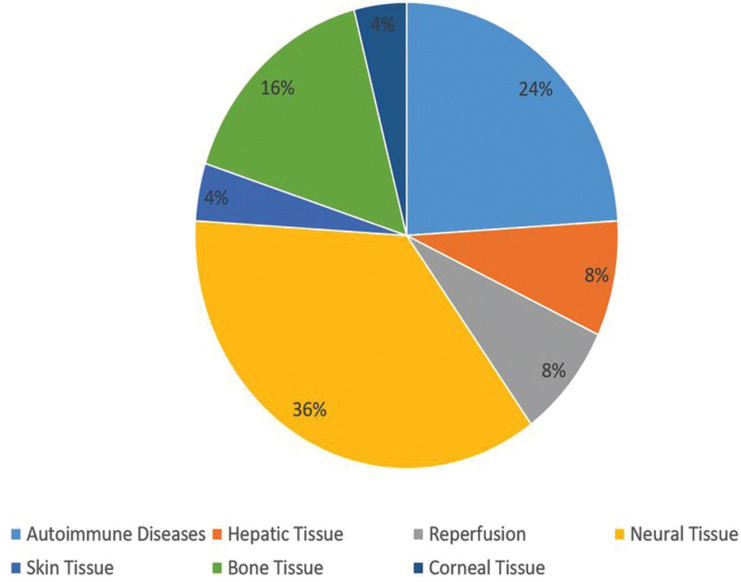
Uses of intraoral MSC.

68% of the total articles included in this review indicate the ability of stem cells from the oral cavity to improve multiple tissues or organs damaged by chemical or physical agents such as: improving the structure and function of the liver with liver cirrhosis (5,6), decrease damage on skin exposed to UV-B rays (7), decrease the damage in organs subjected to heat strokes (8) and cerebral ischemia (9) , allow the formation of bone tissue (10-13) and corneal repair (14), improve motor function (15-18) and the muscle action potentials in defective nerve tissue (19,20), increase the regeneration and survival of ganglion retinal cells (21) and reduce apoptosis of neuronal cells (16). 24% of all articles show therapeutic effects in diseases caused by the immune system, where rheumatoid arthritis is found (22,23), autoimmune encephalomyelitis (24-26), Miastenia Gravis (27) and diabetic polyneuropathy (28,29) (Fig. 3).

## Discussion

According to this review, the most commonly used intraoral MSC donor site is the SHED, being used by 56% of the total number of studies in this review. 28% of the articles included, used a conditioned medium from stem cells of exfoliated temporal teeth (SHED-CM). The 7 articles obtained beneficial effects for the corresponding diseases or injuries in which they were conducting their research. Regarding therapeutic uses, the literature also points out the use of MSC of intraoral origin at the level of different tissues:

1. Bone tissue

The bone tissue lesions are 16% of the total of the studies included in this review, of which 75% performed the transplant of DPSCs and the remaining 25% performed the transplant with DFSCs. Despite having used different scaffolds the four authors agree in their results in having a greater formation of bone compared to the control group, which presented less or no formation of bone tissue (10-13).

2. Nervous tissue

In relation to the therapeutic uses of intraoral MSC, 36% of the total articles used them as cell therapy in nervous tissue, using 33% SHED, 33% SHED-CM and 33% DPSCs. The studies support the use of this type of cells because they come from the neural crest, due to its ability to express neural markers on its surface, its ability to differentiate into neurons under *in vitro* conditions and to protect neurons from cellular apoptosis (8,16,19). Feitosa *et al*. (15) and Nicola *et al.* (16,18) conducted studies with SHED in animals with spinal cord injury. Both authors report improvements in motor functions after the MSC transplant, where Nicola *et al.* (16) it is associated with the decrease of apoptosis of neuronal cells and the maintenance of antiapoptotic factors, which would reduce the damage in this type of lesions, thinking that 90% of the neurons present apoptosis in the first 8 hours after an injury at the level of the spinal cord. In another study of the same author (18) they report that and early transplantation of SHED, in the acute phase of the spinal cord contusion there is an increase of the progenitor cell at 24 hours and reduces the glial scar tissue, so it works as a neuroprotector agent. Asadi-Golsham *et al.* (17) used intraspinal administration of SHED-CM and they also reported functional recovery in chronic spinal injuries. The authors named above do not achieve regeneration of nervous tissue unlike Sugimura-Wakayama *et al.* (19) and Mead *et al.* (21). where the first author reports an increase in myelinated nerve fibers after a lesion in the sciatic nerve in rats using SHED-CM and the second, describes axonal regeneration with an increase in ganglion retinal cells after a lesion occurs in retinal ganglion cells using DPSC. Tsuruta *et al.* (20) also used SHED-CM as a therapy for a nerve lesion model causing dysphagia. They reported that SHED-CM protects the swallowing reflex, reduces pharyngeal residue and promotes axon regeneration.

In cases of diabetic polyneuropathy, Hata *et al.* (28) and Omi *et al.* (29) reports that using DPSC there is a significantly improvement of the motor and sensitive nerve conduction velocity, blood flow, myelin thickness and area of the nerve.

3. Eye tissue and skin

The therapeutic effects on damaged corneas was manifested by 4% of the articles included in this review, where Gomes *et al.* (14) performs the transplant of SHED to rabbit corneas with chemical damage, simulating a total deficiency of limbal stem cells. Clearer corneas were obtained in the groups with MSC transplantation compared to the control groups, where the transparency was greater and with less neovascularization in the group with medium degree damage, this group also showed multiple layers of corneal epithelium with well differentiated cells.

Like the corneal injury, damage to the skin tissue was studied by 4% of the selected articles, which is equivalent to one article. Ueda y Nishino. (7) they carry out an investigation with 24 mice which expose them to UV-B rays and divide them into 3 treatment groups, where they are given a subcutaneous injection with: exfoliated temporary tooth stem cells, serum-free conditioned medium derived from stem cells of temporary teeth exfoliated and a control group. Both treatments performed with exfoliated temporary tooth stem cells reduced wrinkles in number, depth and area, being more effective in the treatment with exfoliated temporary teeth stem cells than the treatment with their serum free conditioned medium.

4. Liver tissue

8% of the selected studies evaluated the therapeutic effects produced by SHED in mice with induced liver cirrhosis, where there is a coincidence in the results obtained in terms of a decrease in liver fibrosis, improvement of liver function and decrease in proinflammatory cytokines. Despite having used different methodologies, where Hirata *et al.* (6) performed intravenous administration of SHED-CM, while Yamaza *et al.* (5) performed a SHED direct transplant into the spleen of the mice.

5. Autoimmune diseases 

24% of the total studies included in this review (6 articles) used MSC from the oral cavity to be used as treatment in autoimmune diseases. Probably the immunomodulatory and immunosuppressive characteristics of MSCs as described above, makes them an attractive cell type for the treatment of autoimmune diseases.

5.1 Rheumatoid Arthritis

Rheumatoid arthritis is a disease that affects the joints of the body, which was studied by Ishikawa *et al.* (23) and Chen *et al.* (22) through the use of SHED-CM and MSC from the gingival tissue respectively, which were injected intravenously in mice. Both authors describe clinical improvement, decreased synovial inflammation, decreased expression of proinflammatory cytokines and a decrease in the destruction of bone and cartilage tissue.

 Ishikawa *et al.* (23) explained that the change in the polarity of macrophages, where the expression of genes related to proinflammatory macrophages (M1) that initiate inflammation and increases the expression of anti-inflammatory macrophages (M2) secreting anti-inflammatory cytokines counteracting M1 cells decreases. which the increase of M2 would favor the treatment of rheumatoid arthritis. While Chen *et al.* (22) explained it by the increase of regulatory T cells (T lymphocytes), which when expressing FoxP3, CD39 and CD73 contribute to the suppression of the immune system.

5.2 Autoimmune encephalomyelitis 

Three articles investigated the therapeutic effects of intraoral MSCs in autoimmune encephalomyelitis where they used mouse models with multiple sclerosis. Trubiani *et al.* (25) and Shimojima *et al.* (26) 

perform intravenous administration with PLSCs or with SHED-CM while Rossato *et al.* (24) performs an intraperitoneal administration with SHED. In all three studies improvements in the clinical activity of the mice are manifested, however,

Shimojima *et al.* (26) and Trubiani *et al.* (25) they also describe a reduction in the demyelinization, in the axonal loss and in the number of infiltration of inflammatory cells, which was not mentioned in the research carried out by Rossato *et al.* (24) probably because he used a different route of administration than the other two authors.

The results of Rossato *et al.* (24) agree with the results of Chen *et al.* (22) described above, where they increase the T lymphocytes expressing FoxP3, which would allow the suppression of the immune system promoting the use of MSC as a treatment for diseases autoimmune

5.3 Miastenia gravis

The only study that used MSC of the dental follicle in autoimmune diseases, was an investigation carried out by Ulusoy *et al.* (27) for the treatment of mice to which myasthenia gravis was induced by immunization, this is a disease that produces weakness in the skeletal muscles. Within the results, there was a decrease in the severity of the disease, lower immunoglobulin levels, suppression of CD11b cells which stimulate phagocytosis, presentation of antigens and aggregation of neutrophils and as several authors have stated in this review, it decreases the production of proinflammatory cytokine (5,6,8,22-24,26,27).

6.Tissue reperfusion

Several studies have investigated the therapeutic effects of mesenchymal stem cells on the reperfusion or ischemia of damaged tissues. This has been explained by the paracrine properties of these cells where they release growth factors that increase angiogenesis, decrease cellular apoptosis and can differentiate into damaged tissue cells. What could explain the results obtained by Tseng *et al.* (8) where by intravenously administering stem cells of temporary teeth exfoliated after heat stroke, there was a decrease in cellular apoptosis, of ischemic markers, hypoxia and brain damage were reduced. Likewise Zhang *et al.* (9) used DPSCs combined with brain-derived neurotrophic factor (BDNF) and observed an improvement at the neurological level in stroke cases that caused cerebral ischemia.

Within the limitations of the results found in this review is the comparison of studies that did not use the same methodologies in terms of the number of cells, the type of administration, the type of scaffolding and the number of passages of the cells used. Due to all the characteristics mentioned above, it is not possible to determine whether the use of MSCs from the oral cavity is a safe method at the extraoral level, without having a standardized protocol. In this review, all the studies found were conducted on animals, without finding studies in humans, so the results obtained are not possible to extrapolate completely to human health. According to the proposal made by Center for Evidence-Based Medicine (CEBM) of Oxford, these studies do not present a level of clinical evidence. Therefore, human studies are required to see the effectiveness of using stem cells from the oral cavity as treatment of diseases or injuries originating in extraoral tissues, however, it is necessary to standardize the protocols and carry out long-term studies before carrying out tests on human beings so as not to compromise their health.

In conclusion, the use of intraoral MSC presents potential therapeutic uses in other tissues of the organism, where at the bone tissue level there is a greater bone formation; at the level of nervous tissue improves motor activity, neuronal apoptosis decreases and nerve regeneration can occur; at the level of cutaneous tissue the folds decrease and at the level of liver tissue improves the structure and liver function. In relation to autoimmune diseases such as rheumatoid arthritis, autoimmune encephalomyelitis and myasthenia gravis, its potential therapeutic utility has been explained by the suppression of the immune system, where the infiltration of inflammatory cells decreases, the expression of proinflammatory cytokines and increases the expression of anti-inflammatory cytokines, which leads to a decrease in tissue destruction. Although the literature does not report adverse events after cell transplantation, the vast majority of articles included did not mention this information, in addition, the follow-up periods In the investigations, they did not exceed 3 months, so there is a lack of studies that evaluate long-term adverse events. In this review all articles included were made in animals, so it is necessary to highlight the lack of literature with a higher level of evidence. Therefore, it is necessary to conduct studies in humans to establish a final conclusion about the use of intraoral MSCs as a treatment in other tissues of the organism, however, a standardized and safe protocol for its use in humans is required.
